# *In Vitro* Anthelmintic Activity of Ethanol Stem Bark Extract of *Albizia ferruginea* (Guill. & Perr.) Benth

**DOI:** 10.1155/2021/6690869

**Published:** 2021-04-27

**Authors:** Miriam Tagoe, Yaw Duah Boakye, Theresah Appiah Agana, Vivian Etsiapa Boamah, Christian Agyare

**Affiliations:** Department of Pharmaceutics, Faculty of Pharmacy and Pharmaceutical Sciences, Kwame Nkrumah University of Science and Technology, Kumasi, Ghana

## Abstract

*Albizia ferruginea* (Guill. & Perr.) Benth bark is used in the traditional medicine as a vermifuge. This study sought to determine the anthelmintic activity of the stem bark extract of *Albizia ferruginea*. The powdered *A. ferruginea* stem bark was extracted with ethanol. Phytochemical screening was carried out on *A. ferruginea* ethanol extract (AFE) and then screened for its anthelmintic property against *Pheretima posthuma* and *Haemonchus contortus* using the adult motility assay. The effect of AFE and its fractions on the anthelminthic activity of mebendazole and albendazole were also determined using the adult worm (*P. posthuma*) motility assay. AFE showed a dose-dependent anthelmintic activity against *P. posthuma* and *H. contortus*. The least concentration of AFE (0.5 mg/mL) paralyzed and killed *P. posthuma* within 272.50 ± 12.42 min and 354.50 ± 5.06 min of exposure, respectively. AFE at the least test concentration (0.14 mg/mL) caused paralysis and induced death of *H. contortus,* after at 63.50 ± 2.98 and 254.96 ± 2.44 min of exposure, respectively. AFE extract at 0.25 and 0.125 mg/mL increased the paralytic and helminthicidal activities of albendazole. The paralytic and helminthicidal activities of mebendazole were reduced when combined with AFE (0.25 and 0.125 mg/mL). Among the three fractions obtained from AFE, the methanol fraction showed the highest anthelmintic activity. The methanol fraction at 0.5 mg/mL caused paralysis after 69.90 ± 0.15 min and death of worm after 92.53 ± 0.74 min of exposure. The petroleum ether and ethyl acetate fractions showed relatively low anthelmintic activity. Phytochemical screening of AFE revealed the presence of tannins, saponins, glycosides, alkaloids, and coumarins. The results from this study show that *A. ferruginea* possesses anthelmintic activity which gives credence to its folkloric use.

## 1. Introduction

Helminthiasis occurs when there is an infestation of part of the body with worms. These worms are normally found in the digestive tract but may also burrow into the liver and sometimes other organs [1]. Helminthiasis occurs worldwide, especially in tropical and subtropical countries where poverty is high [2]. The World Health Organization reports that over 1.5 billion people are infected with one or more soil-transmitted helminths, notably, hookworm, *Trichuris trichiura*, and *Ascaris lumbricoides* [3]. Approximately, a quarter of the total population in Sub-Saharan Africa is infected with one or more helminths [4] with most West African countries having a prevalence greater than 70% [5]. Nematodes are the most common among all helminths [4]. In areas where prevalence is high, one can be infected simultaneously with more than one type of helminth [6]. The neglected tropical diseases, of which helminth infections form a part, cause about 534,000 deaths every year and a disease burden of 57 million disability-adjusted life–years (DALYs) [7]. Intestinal worms such as *Haemonchus contortus* have been found to have developed resistance to some anthelmintics, namely, levamisole, albendazole, and closantel [8]. Apart from the development of anthelmintic resistance [9, 10], most of the drugs used to treat these worm possess some common side effects such as nausea, vomiting, abdominal pain, and fall in blood pressure in humans [11], and in livestock, coupled with the fact that many farmers in the developing countries are not able to afford synthetic anthelmintics to control the spread [12]. Again, the issue of anthelmintic resistance in humans has become a matter of concern due to the evidence of resistance in livestock [13], thus posing a serious threat to the production of livestock in developing countries. Therefore, there is the need to develop other alternatives for control of helminth infestations [14]. Medicinal plants are among the natural products being explored for their anthelmintic properties [12, 15–17].

*Albizia ferruginea* (Guill. & Perr.) Benth belongs to the family Fabaceae. It is usually found in woodland, lowland rainforest, and scrub vegetation. It is a perennial medicinal plant widely used in Africa [18]. The bark of *A*. *ferruginea* is used in traditional medicine to treat dysentery, bronchial infections, and pain caused by fever. In central Cameroon, inhabitants use leaves maceration as a purgative in children to treat intestinal disorders [19]. Secondary metabolites such as flavonoids [20], terpenoids, saponins, sterols, and tannins have been identified in *Albizia* species [18]. Although *A. ferruginea* has been reported for its antioxidant [21], anti-inflammatory [22], and antimicrobial activities [18], very little research have been carried out on its anthelminthic activities. The stem bark of *A. ferruginea* may have a promising anthelmintic activity due to its traditional usage as a purgative and a vermifuge [23]. This study, therefore, evaluated *Albizia ferruginea* for its anthelmintic properties.

## 2. Materials and Methods

### 2.1. Collection of Stem Bark of *A. ferruginea*

The stem bark of *A. ferruginea* was collected from Kwahu Asasraka (6.627092,-0.692874) in the Eastern Region of Ghana in January 2018. The plant material was identified and authenticated by Dr. George Henry Sam, a lecturer at the Department of Herbal Medicine, Faculty of Pharmacy and Pharmaceutical Sciences, KNUST, Kumasi, Ghana. A voucher specimen with the number KNUST/HMI/2018/SB007 has been kept at the herbarium of the Department of Herbal Medicine, KNUST. The sample was dried in shade for seven days to obtain uniform dry weight. Ten kilograms of the dried sample was then milled and stored at 25°C in glass containers for further investigations.

### 2.2. Preparation of Ethanol Extract of *A. ferruginea*

Powdered plant material (150 g) of *A. ferruginea* bark was weighed and suspended in 1600 mL of ethanol (UK Chemicals, Kumasi, Ghana) and shaken intermittently for 3 days at room temperature. The suspension was then filtered using Whatman filter paper number 1 (Sigma-Aldrich, London, UK), and the filtrate was concentrated using a rotary evaporator (Genser Scientific Instruments, Germany) at 40°C under reduced pressure. The extract was kept at 4°C in the refrigerator until needed. The ethanol stem bark extract of *A. ferruginea* was coded as AFE, and its percentage yield was calculated to be 9.8% *w*/*w*.

### 2.3. Phytochemical Screening

Phytochemical screening of ethanol extract of *A. ferruginea* was carried out to detect the presence or otherwise of secondary metabolites such as tannins, alkaloids, and flavonoids, following standard procedures [24, 25].

### 2.4. Fractionation of AFE Extract Using Column Chromatography

Silica gel (70 to 230 mesh, Sigma-Aldrich, London, UK) was used as the stationary phase. Fifteen (15.0) grams of blank silica was packed into a dried glass column (50 mm × 42 cm) (Fisher Scientific GmbH, Schwerte, Germany). Afterwards, 136 g of the AFE was reconstituted in 25 mL of 70% ethanol and adsorbed unto 10 g of silica gel. The slurry obtained was dried thoroughly at room temperature. The mixture obtained was then loaded on top of a previously packed blank silica within the column. The mobile phases, petroleum ether, ethyl acetate and methanol (UK Chemicals, Kumasi, Ghana), were used. Elution began with absolute petroleum ether, and when the eluate was colourless, ethyl acetate was used followed by methanol. The fractions were collected and bulked according to their TLC profiles [26].

### 2.5. Test Organisms

*Pheretima posthuma* commonly known as adult Indian earthworms have an anatomical and physiological semblance to the human intestinal roundworms *Ascaris lumbricoides* [27]. Adult *P. posthuma* (earthworms) were collected from the Wiwi River, behind the Department of Theoretical and Applied Biology, College of Science, Kwame Nkrumah University of Science and Technology (KNUST), Kumasi, Ghana. The earthworms were washed with Ringer's lactate solution to remove soil debris.

*Haemonchus contortus* is a highly pathogenic parasite of small ruminants [28]. This worm feeds on the blood of ruminants and grows well in warm temperate, tropical, and subtropical regions [29]. *Haemonchus contortus* worms were collected from the intestines of slaughtered cows at the Tamale Abattoir, Northern Region of Ghana, with the help of the Veterinary Officers and kept in Ringer's lactate solution for transportation. The length of *P. posthuma* and *H. contortus* measured were within the ranges of 5.0 to 7.0 cm, and 4.0 to 6.0 cm, respectively.

### 2.6. Determination of Anthelmintic Activities

The anthelmintic activity against *P. posthuma* was determined using the adult motility assay described by Chander et al. [17]. Nine concentrations (32, 16, 8, 4, 2, 1, 0.5, 0.25, and 0.125 mg/mL) of AFE and its fractions (methanol, ethyl acetate, and petroleum ether) were prepared. Adult earthworms 5 to 7 cm in length (*P. posthuma*) were aseptically transferred into appropriately labelled petri dishes (Fisher Scientific GmbH, Schwerte, Germany) containing different concentrations of AFE and AFP. The worms were then observed over a maximum of 8 h for paralysis and death. Paralysis was said to occur when no movement of any part was observed except when the worms were vigorously shaken. Death was ascertained when the worms neither moved when shaken vigorously nor revived when placed in Ringer's lactate solution (B. Braun Medical Ltd., UK) and sometimes followed by fading away of their body colour. The positive control group was placed in petri dishes containing albendazole (Sigma-Aldrich, London, UK) (10, 5, 2.5, 1.25, and 0.625 mg/mL). Three (3) worms of about the same size per petri dish were used. They were observed for their motility and the time taken for paralysis and death of worms. The above procedure was carried out in triplicates.

For *H. contortus*, its anthelmintic activity was conducted using the adult motility assay described by Jabbar et al. [12]. Mature live *H. contortus* worms were washed in Ringer's lactate solution. Seven groups consisting of three worms each were used for this assay. The worms in the various groups were exposed to the following treatments, respectively: 0.57, 0.285, 0.14, 0.07, and 0.035 mg/mL of AFE, 0.5 mg/mL albendazole (positive control), and Ringer's lactate (negative control). The worms were observed over an 8 h period for paralysis and death. Paralysis was said to occur when no movement of any part was observed except when the worms were vigorously shaken. Death was ascertained when the worms neither moved when shaken vigorously nor revived when placed in Ringers lactate solution and sometimes followed by fading away of their body colour.

### 2.7. Determination of Resistance Modifying Activity of AFE Extract against *P. posthuma*

The effect of the ethanol extract of *A*. *ferruginea* on the activity of albendazole (Sigma-Aldrich, London, UK) against *P. posthuma* worms was determined. Two subactivity concentrations of the AFE extract (0.25 and 0.125 mg/mL) and 10 mg/mL stock solution of albendazole were used. The albendazole stock solution was serially diluted to obtain the following concentrations: 5, 2.5, 1.25, and 0.625 mg/mL. A volume of 50 mL of each concentration was transferred into appropriately labelled petri dishes (three petri dishes per concentration). Three adult *P*. *posthuma* worms of lengths ranging between 5 and 7 cm were placed into each of the labelled petri dishes filled with the respective concentrations of albendazole. The set up was observed for 8 hours for the time of paralysis and death. The procedure was performed in triplicates. The above procedure was repeated using mebendazole (Sigma-Aldrich, London, UK) [30].

### 2.8. Statistical Analysis

Data were analysed with GraphPad Prism version 8.0 for Windows (Graph Pad Software Inc., San Diego, CA, USA). One-way ANOVA followed by Dunnett's *post hoc* test was used to analyse data obtained for anthelmintic studies.

## 3. Results

### 3.1. Phytochemical Screening of AFE Extract

Phytochemical screening was conducted to determine the probable phytochemicals in AFE. Phytochemical screening of the AFE revealed that tannins, saponins, glycosides, alkaloids, and coumarins were present but flavonoids were absent (Table 1).

### 3.2. Determination of Anthelmintic Assay Using *P. posthuma*

**Figure 1:**
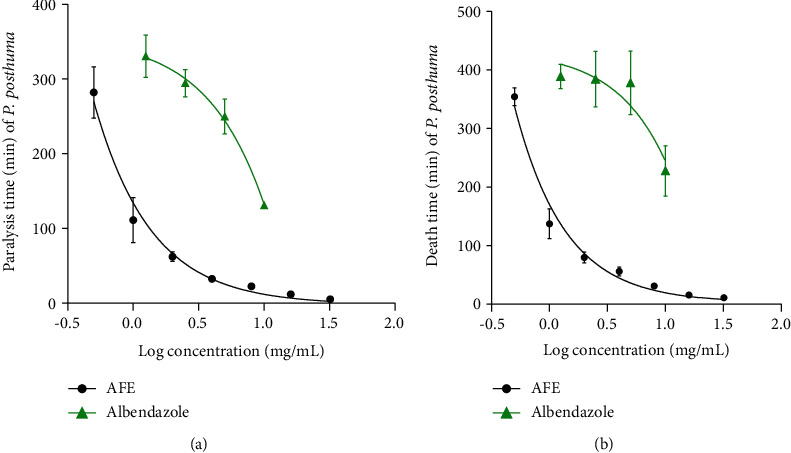
Effect of AFE and albendazole on paralysis and death of *P. posthuma.* (a) Paralysis time. (b) Death time. No paralysis and death was observed after the maximum time of exposure (8 hours) in the negative control group (Ringer's lactate solution). AFE: ethanol stem bark extract of *A. ferruginea*.

At the highest concentration of 32 mg/mL, AFE paralyzed and killed all worms within 5.67 ± 0.67 and 8.82 ± 0.82 min of exposure, respectively. At the least concentration of 0.5 mg/mL, AFE paralyzed and killed all worms within 272.50 ± 12.42 min and 354.50 ± 5.06 min of exposure, respectively (Figure 1(a)). Albendazole (10 mg/mL) paralyzed worms within 130.72 ± 1.50 min and killed the worms within 232.15 ± 1.29 min of exposure. At 1.25 mg/mL, albendazole paralyzed and killed worms within 330.60 ± 9.47 min and 389.16 ± 7.06 min of exposure, respectively (Figure 1(b)).

### 3.3. Determination of Anthelmintic Assay Using *H. contortus*

**Figure 2:**
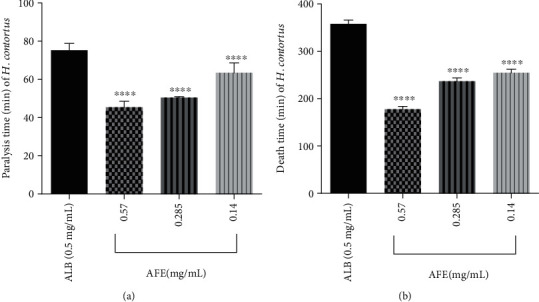
Effect of AFE on paralysis and death time of *H. contortus.* (a) Paralysis time. (b) Death time. ALB: albendazole; *n* = 9, values are mean ± SEM; ^∗∗∗∗^*p* < 0.0001, compared to the control (one-way ANOVA followed by Dunnett's *post hoc* test). No paralysis was observed after maximum time of exposure (8 hours) in the negative control group (Ringer's lactate solution).

AFE showed a dose-dependent activity against *H. contortus.* At concentrations of 0.14, 0.285, and 0.57 mg/mL, AFE significantly (*p* value < 0.0001) caused paralysis of worms at 63.50 ± 2.98, 55.75 ± 1.41, and 46.93 ± 0.85 min, respectively, compared to the negative control (Ringer's lactate solution) which showed no paralysis after the maximum time (8 h) of exposure (Figure 2(a)). Concentrations of AFE at 0.14, 0.285, and 0.57 mg/mL significantly (*p* value < 0.0001) caused death of worms at 254.96 ± 2.44 min, 236.979 ± 2.272 min, and 177.933 ± 1.929 min of exposure, respectively (Figure 2(b)), and this was significant (*p* < 0.0001) when compared with the negative control group. Albendazole (0.5 mg/mL) caused paralysis and death of the worms at 75.26 ± 2.00 and 258.09 ± 2.84 min of exposure, respectively (Figure 2).

### 3.4. Influence of AFE Extract on Anthelmintic Activity of Albendazole

**Figure 3:**
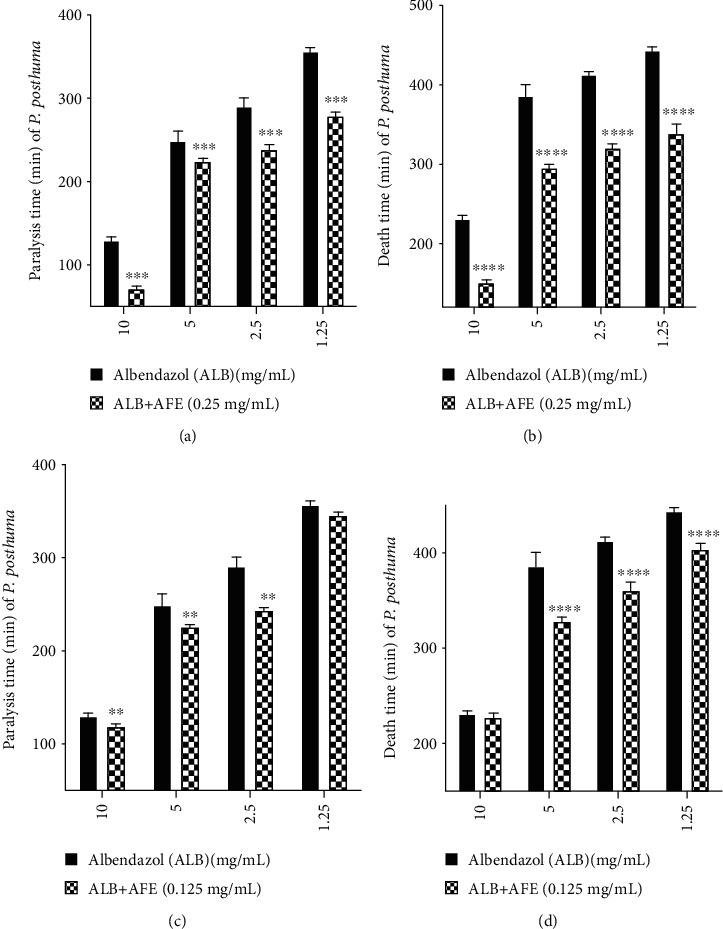
Effect of AFE on paralytic and helminthicidal activity of albendazole. (a, b) Effect of 0.25 mg/mL AFE on albendazole. (c, d) Effect of 0.125 mg/mL AFE on albendazole. ALB: albendazole; AFE: *A. ferruginea* ethanol stem bark extract; ^∗∗∗∗^*p* < 0.0001, ^∗∗∗^*p* < 0.001, and ^∗∗^*p* < 0.01 compared to control (one-way ANOVA followed by Dunnett's *post hoc* test).

Albendazole alone at 10 mg/mL paralyzed all worms after 130.721 ± 1.499 min of exposure. AFE (0.25 mg/mL) combined with 10 mg/mL albendazole significantly (*p* < 0.001) reduced the paralysis time. When 10 mg/mL albendazole was combined with 0.25 mg/mL AFE, the worms were paralyzed after 72.296 ± 0.777 min of exposure. Similar effects were observed at lower concentrations of albendazole (5, 2.5, and 1.25 mg/mL) combined separately with 0.25 mg/mL AFE (Figure 3(a)).

Albendazole (10 mg/mL) killed all worms after 232.147 ± 1.293 min of exposure. When 0.25 mg/mL AFE was combined with 10 mg/mL albendazole, it significantly (*p* < 0.0001) reduced the death time. Combination of 10 mg/mL albendazole with 0.25 mg/mL AFE killed the worms after 151.752 ± 0.960 min of exposure. Similar effects were observed at lower concentrations of albendazole (5, 2.5, and 1.25 mg/mL) combined separately with 0.25 mg/mL AFE (Figure 3(b)).

Albendazole (10 mg/mL) paralyzed all worms after 130.721 ± 1.499 min of exposure. When albendazole (10 mg/mL) was also combined with 0.125 mg/mL AFE, the worms were paralyzed after 119.712 ± 0.713 min of exposure which showed significant (*p* < 0.01) reduction in paralysis time. Similar effects were observed when lower concentrations of albendazole (5 and 2.5 mg/mL) were combined separately with 0.125 mg/mL AFE. Albendazole (1.25 mg/mL) paralyzed all worms after 357.7 ± 2.975 min of exposure. When 1.25 mg/mL albendazole was combined with 0.125 mg/mL AFE, paralysis time (346.2 ± 1.729 min) was reduced as compared to albendazole (1.25 mg/mL) but this reduction in time was not significant (Figure 3(c)).

Albendazole alone at 10 mg/mL killed all worms after 232.147 ± 1.293 min of exposure. When 10 mg/mL albendazole was combined with 0.125 mg/mL AFE, the worms were killed after 224.954 ± 1.164 min of exposure, significantly (*p* < 0.0001) reducing the death time. Similar effects were observed at lower concentrations of albendazole (5, 2.5, and 1.25 mg/mL) combined separately with 0.125 mg/mL AFE (Figure 3(d)).

### 3.5. Influence of AFE on Anthelmintic Activity of Mebendazole

**Figure 4:**
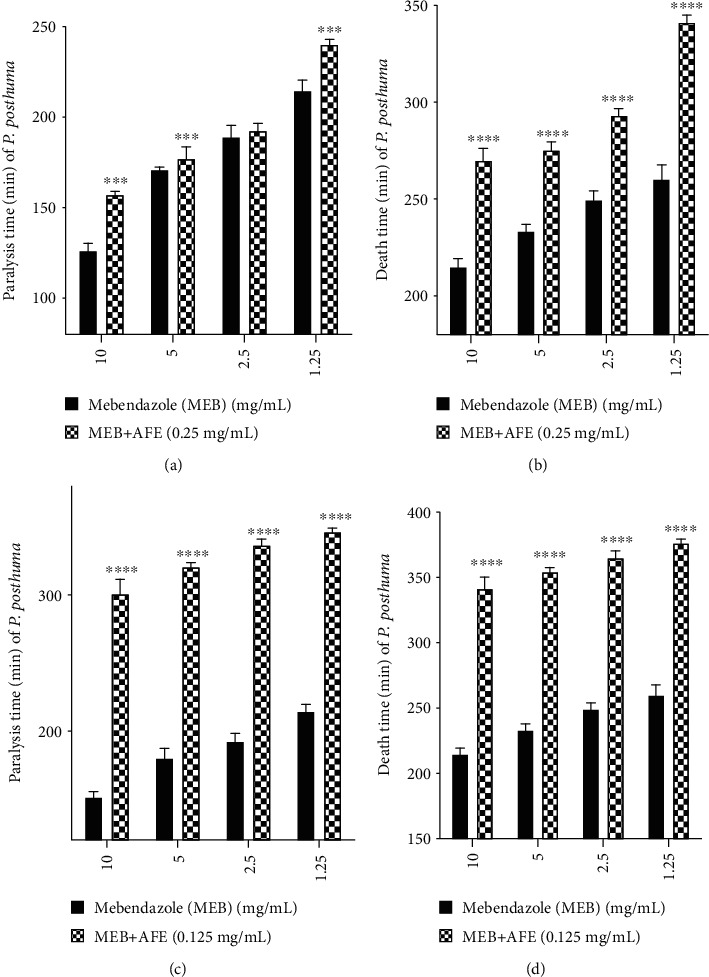
Effect of AFE on paralytic and helminthicidal activity of mebendazole. (a, b) Effect of 0.25 mg/mL AFE on mebendazole.(c, d) Effect of 0.125 mg/mL AFE on mebendazole. MEB: mebendazole; AFE: *A. ferruginea* ethanol stem bark extract; ^∗∗∗∗^*p* < 0.0001, ^∗∗∗^*p* < 0.001 compared to the control (one-way ANOVA followed by Dunnett's *post hoc* test).

Mebendazole alone at 10 mg/mL caused paralysis within 150.653 ± 0.735 min of exposure. When combined with AFE (0.25 mg/mL), paralysis time increased to 157.288 ± 0.572 min of exposure, significantly (*p* < 0.001) inhibiting the activity of mebendazole. Similar effects were observed when lower concentrations (5, 2.5, 1.25, and 0.625 mg/mL) of mebendazole were combined with 0.25 mg/mL of AFE (Figure 4(a)).

Mebendazole alone at 10 mg/mL killed worms after 212.3967 ± 0.974 min of exposure. When combined with AFE (0.25 mg/mL), the worms were killed after 260.963 ± 0.981 min of exposure, thereby significantly (*p* < 0.0001) inhibiting the activity of mebendazole. Similar effects were observed when lower concentrations of mebendazole (5, 2.5, 1.25, and 0.625 mg/mL) were combined with 0.25 mg/mL AFE extract Figure 4(b).

Mebendazole alone at 10 mg/mL caused paralysis within 150.653 ± 0.735 min of exposure. Addition of AFE (0.125 mg/mL) caused paralysis after 298.273 ± 1.157 min of exposure, thereby significantly (*p* < 0.0001) inhibiting the activity of mebendazole. Similar effects were observed when different concentrations of mebendazole (5, 2.5, 1.25, and 0.625 mg/mL) were combined with 0.125 mg/mL AFE Figure 4(c).

Mebendazole alone at 10 mg/mL killed worms after 212.3967 ± 0.974 min of exposure. When mebendazole (10 mg/mL) was combined with AFE (0.125 mg/mL), the worms were killed within 337.375 ± 1.267 min, significantly (*p* < 0.0001) inhibiting the activity of mebendazole. Similar effects were observed when lower concentrations of mebendazole (5, 2.5, 1.25, and 0.625 mg/mL) were combined with 0.125 mg/mL AFE (Figure 4(d)).

### 3.6. Anthelmintic Activity of AFE Fractions against *P. posthuma*

**Figure 5:**
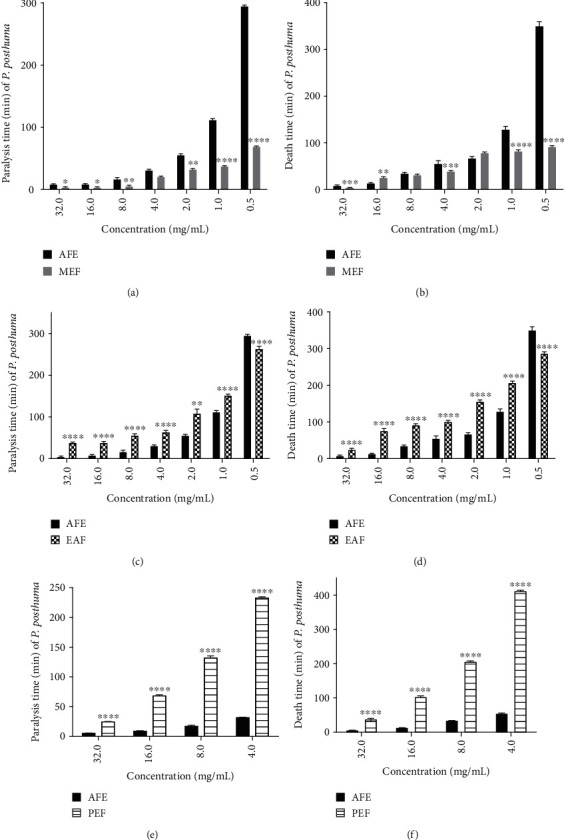
Comparison of the effect of AFE and its fractions (methanol, ethyl acetate, and petroleum ether fractions) on paralysis and death time of *P. posthuma.* (a, b) Paralysis and death time of methanol fraction, respectively. (c, d) Paralysis and death time of ethyl acetate fraction, respectively. (e, f) Paralysis and death time of petroleum ether fraction, respectively; ^∗∗∗∗^*p* < 0.0001, ^∗∗∗^*p* < 0.001, ^∗∗^*p* < 0.01, and ^∗^*p* < 0.05 compared to control (one-way ANOVA followed by Dunnett's *post hoc* test). AFE: *A. ferruginea* ethanol stem bark extract; MEF: methanol fraction of AFE; EAF: ethyl acetate fraction of AFE; PEF: petroleum ether fraction of AFE.

All three fractions of AFE were active and expressed a concentration-dependent activity against *P. posthuma* (Figure 5). The methanol fraction at its highest and lowest concentrations (32 and 0.5 mg/mL) caused paralysis after 4.13 ± 0.12 and 69.90 ± 0.15 min, and death of worm after 4.13 ± 0.12 and 92.53 ± 0.74 min of exposure, respectively (Figures 5(a) and 5(b)). The ethyl acetate fraction at its highest and lowest concentrations (32 and 0.5 mg/mL) caused paralysis after 38.90 ± 0.33 and 264.24 ± 2.90 min, and death of worm after 25.25 ± 1.52 and 287.33 ± 3.05 min of exposure, respectively (Figures 5(c) and 5(d)). The petroleum ether fraction at its highest and lowest concentrations (32 and 0.5 mg/mL) caused paralysis after 24.60 ± 0.40 and 233.07 ± 1.73 min, and death of worm after 39.64 ± 0.60 and 413.53 ± 0.774 min of exposure, respectively (Figures 5(e) and 5(f)).

## 4. Discussion

Plants possess various medicinal properties due to the phytochemicals they produce during secondary vegetal metabolism [31, 32]. In this study, the ethanol stem bark extract of *A. ferruginea* was found to contain tannins, saponins, glycosides, alkaloids, and coumarins (Table 1). This finding is in agreement with a report by Agyare et al. [18] who found that the ethyl alcohol extract of leaves and stem bark of *A. ferruginea* contained tannins and saponins.

The anthelmintic activity of AFE (Figures 1 and 2) may be due to the presence of phytochemicals such as saponins, glycosides, and alkaloids. This is because it has been reported that secondary metabolites such as alkaloids and saponins possess anthelmintic activity [33–35]. Alkaloids which act as antioxidants reduce the generation of nitrate, which is known to interfere with local homeostasis which is necessary for helminth development [36]. Therefore, a reduction in nitrate generation by alkaloids in the extract probably may lead to the death of helminths.

*Pheretima posthuma* was used in this study due to its anatomic and physiological resemblance to the human intestinal roundworm, *Ascaris lumbricoides* [37]*. Haemonchus contortus* (strongylid nematode) which is a gastrointestinal parasitic nematode of small ruminants was also used because it is closely related to the human hookworm [38]. AFE showed dose-dependent paralytic and lethal effects on both *P. posthuma* and *H. contortus* (Figures 1 and 2). Studies have shown that a plant that exhibits anthelmintic activity against *P. posthuma* may show anthelmintic activity against *H. contortus* as well [39, 40]. Since *H. contortus* is closely related to the human hookworm species, AFE may be active against the human hookworm as well [38]. Khan et al. (2010) reported that the methanol extract of *Albizia lebbeck*, an *Albizia* species, possesses anthelmintic activity against *H. contortus.* This supports the findings of this study and gives credence to the folkloric use of *Albizia* species as anthelmintics [19].

Plants have been known to enhance or inhibit the activity of known anthelmintic agents [30]. The anthelmintic activity of albendazole against *P. posthuma* was significantly (*p* < 0.0001, *p* < 0.001, and *p* < 0.01) enhanced in the presence of subactivity concentrations of AFE (Figure 3). This may involve enhanced disruption of the integrity of the helminth tegument, inhibition of motility, and reduction in glucose uptake by the worm [41] leading to the paralysis and eventually death of the worm. In a similar study [30], it was reported that the enhanced anthelmintic activity of albendazole may be due to the presence of secondary metabolites in the extract that increased drug uptake into the organism, making the drugs more available to the binding sites and eventually potentiating the activity of the anthelmintic drug.

AFE when combined with mebendazole significantly inhibited the paralytic and lethal activity of mebendazole (Figure 4). The exact mechanism of interaction between the drug and herb is not yet known [42]. However, some extracts may contain antagonistic compounds which may likely reduce the efficacy of a pharmaceutical drug [43, 44]. It has been reported that some phytochemicals can form complexes with antimicrobial agents which can lead to reduced absorption, decreased affinity to the binding site, and subsequent loss of activity [45].

Seelinger et al. [46] observed that fractionation of plant extracts may lead to the isolation and separation of distinct desired properties of a plant. Methanol, ethyl acetate, and petroleum ether fractions of AFE were active against *P. posthuma*, and out of the three fractions, methanol fraction had a better anthelmintic activity (Figure 5). The methanol fraction also had better anthelmintic activity than the AFE (Figures 5(a) and 5(b)). Abu-Lafi et al. [47] reported that fractions may have better activity than whole extracts. The secondary metabolites present in *A. ferruginea* may be very polar since methanol is more polar than ethanol and most of the phytochemicals might have partitioned into it [48, 49]. The AFE had better anthelmintic activity compared to the petroleum ether and ethyl acetate fractions. This could be due to the various secondary metabolites within the crude AFE that acted synergistically for its better anthelmintic activity [46].

## 5. Conclusions

The ethanol stem bark extract of *A. ferruginea* (AFE) possesses anthelmintic activity against *P. posthuma* and *H. contortus*. The methanol, ethyl acetate, and petroleum ether fractions of AFE also exhibited anthelmintic activity against *P. posthuma*. AFE significantly improved the paralytic and lethal effects of albendazole but inhibited the paralytic and lethal activity of mebendazole. Phytochemical screening performed on AFE revealed that it contains tannins, saponins, glycosides, alkaloids, and coumarins.

## Figures and Tables

**Table 1:** Phytochemical screening of AFE.

Secondary metabolites	AFE
Tannins	+
Saponins	+
Glycosides	+
Alkaloids	+
Flavanoids	−
Coumarins	+

+: the presence of secondary metabolite; −: absence of secondary metabolite.

## Data Availability

All data obtained from this study are within the manuscript.
